# Microwave–Ultrasound-Assisted Extraction Coupled with Natural Deep Eutectic Solvent Enables High-Yield, Low-Solvent Recovery of Curcumin from *Curcuma longa* L.

**DOI:** 10.3390/pharmaceutics17070818

**Published:** 2025-06-24

**Authors:** Muhammad Sahlan, Desy Rosarina, Hasna Farida Ratna Suminar, Yoga Diatama Pohan, Ibnu Maulana Hidayatullah, Dimas Rafi Narawangsa, Dwini Normayulisa Putri, Eka Sari, Meka Saima Perdani, Yudha Gusti Wibowo, Heri Hermansyah

**Affiliations:** 1 Department of Chemical Engineering, Faculty of Engineering, Universitas Indonesia, Kampus UI Depok, Depok 16424, West Java, Indonesia; desy.rosarina@ui.ac.id (D.R.); hasna.farida@ui.ac.id (H.F.R.S.); yoga.diatama@ui.ac.id (Y.D.P.); ibnu.maulana.h@che.ui.ac.id (I.M.H.); dimas.rafi@ui.ac.id (D.R.N.); 2 Research Center for Biomedical Engineering, Faculty of Engineering, Universitas Indonesia, Kampus UI Depok, Depok 16424, West Java, Indonesia; 3 Department of Industrial Engineering, Faculty of Engineering, Universitas Muhammadiyah Tangerang, Tangerang 15118, Banten, Indonesia; 4 Research Center for Biomass Valorizations, Universitas Indonesia, Kampus UI Depok, Depok 16424, West Java, Indonesia; 5 Research Center for Biomass and Bioproducts, National Research and Innovation Agency, Cibinong 16911, West Java, Indonesia; dwini014@brin.go.id; 6 Chemical Engineering, Engineering Faculty, Sultan Ageng Tirtayasa University, Cilegon 42434, Banten, Indonesia; ekasari@untirta.ac.id; 7 Bioengineering & Biomedical Engineering, Research Centre CoE, Engineering Faculty, Sultan Ageng Tirtayasa University, Cilegon 42434, Banten, Indonesia; 8 Department of Chemical Engineering, Faculty of Engineering, Universitas Singaperbangsa Karawang, Karawang 41361, West Java, Indonesia; meka.perdani@ft.unsika.ac.id; 9 Center for Green and Sustainable Materials, Institut Teknologi Sumatera, Terusan Ryacudu, Way Hui, Jati Agung, Bandar Lampung 35365, Lampung Selatan, Indonesia; yudha.wibowo@ta.itera.ac.id

**Keywords:** curcumin, *Curcuma longa* L., NADES, MUAE, low-solvent extraction, sustainable process

## Abstract

**Background/Objectives**: Solvent-intensive methods are traditionally required to extract curcumin, a potent bioactive compound from *Curcuma longa*, raising environmental and safety concerns. **Methods**: This study introduces an efficient and scalable extraction approach using microwave–ultrasound-assisted extraction (MUAE) combined with a natural deep eutectic solvent (NADES) composed of choline chloride and lactic acid. Process parameters, including solvent water content (20–30% *v*/*v*) and solid loading (4–8% *w*/*v*), were optimized using response surface methodology (RSM) to enhance curcumin yield. **Results:** Under optimal conditions (20% water content and 8% solid loading), the MUAE method achieved a curcumin content of 40.72 ± 1.21 mg/g, representing a 14.36% improvement over conventional ultrasound-assisted extraction (UAE), while reducing solvent usage by 50%. The quadratic model demonstrated excellent predictive capability, with an R^2^ value of 0.98. In addition, anti-solvent precipitation using water increased curcuminoid purity from 0.31% to 20.54%, with a recovery rate of 21.49%. **Conclusions:** Mechanistic analysis revealed that microwave-induced cell disruption, ultrasound cavitation, and the modulation of NADES viscosity contributed synergistically to the enhanced extraction performance. This study is the first to combine MUAE with NADES for optimized curcumin extraction, delivering both high yield and reduced solvent consumption. The proposed method offers a sustainable and industrially relevant alternative for curcumin recovery in the food, nutraceutical, and pharmaceutical sectors.

## 1. Introduction

Turmeric (*Curcuma longa* L.), a rhizomatous plant from the Zingiberaceae family, has long been recognized for its culinary, agri-food, and medicinal applications [1,2,3]. As the primary bioactive constituent of turmeric, curcumin exhibits a broad spectrum of pharmacological activities, including antioxidant [4], anti-inflammatory [5], antimicrobial [6], anticancer [7], and neuroprotective effects [8,9]. Given its therapeutic potential, curcumin has attracted significant interest from the food, nutraceutical, and pharmaceutical industries, fueling the demand for efficient, sustainable, and scalable extraction techniques.

Conventional extraction methods, such as maceration, Soxhlet extraction, and reflux, require large volumes of organic solvents, extended processing times, and high energy input, often leading to degradation of thermosensitive compounds and generation of hazardous waste. In response, recent efforts have focused on utilization of low-solvent extraction technologies that align with the principles of sustainability and reduce environmental impact. Among them, ultrasound-assisted extraction (UAE) and microwave-assisted extraction (MAE) have shown promise in enhancing extraction efficiency while minimizing solvent and energy consumption [10]. However, most studies to date have applied these techniques independently and often still rely on conventional organic solvents such as ethanol, acetone, or methanol, raising concerns about solvent toxicity, flammability, and regulatory acceptability in food and pharmaceutical applications.

A particularly promising alternative to organic solvents is the use of Natural Deep Eutectic Solvents (NADES) with low-toxicity, biodegradable solvents composed of naturally derived hydrogen bond donors and acceptors [11,12,13]. In several studies, NADES have demonstrated excellent solubilizing capacity for both hydrophilic and hydrophobic bioactives, including curcumin, due to their tunable polarity and ability to form strong hydrogen bonding interactions [14,15,16,17,18]. Despite their advantages, the integration of NADES with process-intensified techniques, such as MAE and UAE, remain underexplored. Only a limited number of studies have investigated the synergistic combination of microwave and ultrasound energy with NADES for the extraction of curcumin, and none have comprehensively optimized the process variables needed to minimize solvent usage while maximizing yield and purity.

Only a few studies have investigated the synergistic integration of microwave and ultrasound-assisted extraction (MUAE) with NADES for the recovery of curcumin. For instance, a study examined microwave-assisted extraction of curcumin using NADES; it achieved moderate yield improvements, but it required longer extraction times and higher solvent consumption, and did not address solvent recyclability [19]. Another previous study used a NADES composed of nipa palm syrup and vinegar with MAE, yielding 43.04 mg/g curcumin in 51 s. However, ultrasound energy was not applied, and solvent economy was not optimized [20]. In line with this, another study extracted 89.87 mg/g curcuminoids using microwave-assisted DES extraction with choline chloride–citric acid DES, requiring high solvent-to-solid ratios and additional anti-solvent steps for recovery [19]; an additional study optimized MAE using five NADES formulations and reported improved antioxidant capacity, but the maximum curcumin yield was not specified beyond relative performance compared to 80% methanol [21].

In contrast, the present study introduces a fully optimized MUAE–NADES platform for curcumin extraction. It investigated 20 NADES systems and found choline chloride-based solvents provided curcumin yields of 30.73–31.70 mg/g, outperforming water. However, reduced efficiency was observed at higher solid loadings, and solvent/water optimization was not performed [22]. This system integrates the benefits of both microwave and ultrasound energy namely, rapid cell wall disruption and enhanced mass transfer with the environmentally friendly properties of NADES. Moreover, this synergistic method has also shown promise in extracting other classes of polyphenols. A study demonstrated that MUAE–NADES improved pectin and polyphenol recovery from dragon fruit peels, achieving significantly higher antioxidant activity and reducing processing time. This highlights MUAE’s potential for broader polyphenolic applications [23]. Similarly, utilization of ultrasonic-microwave-assisted NADES (UMAE–NADES) to extract anthocyanins from *Aronia melanocarpa*, reaching a purity of 448.87 mg/g and greater antioxidant activity than conventional methods [24].

To address this gap, the present study proposes a MUAE system using a NADES composed of choline chloride and lactic acid for the efficient, sustainable extraction of curcumin from *Curcuma longa.* Unlike previous approaches, this work not only integrates both microwave and ultrasound technologies but also optimizes the solvent composition (water content in NADES) and solid loading using response surface methodology (RSM). Moreover, it evaluates the solvent reduction potential, recovery, and purity of curcuminoids via water-based precipitation, and the overall suitability of the system utilized for low-solvent and scalable industrial application. This study is the first to demonstrate that the MUAE–NADES system can deliver higher curcumin yields with significantly reduced solvent consumption while eliminating the use of organic solvents entirely. The findings contribute to advancing low-solvent extraction and provide a valuable foundation for the development of cleaner, safer, and more cost-effective phytochemical recovery processes.

## 2. Materials and Methods

### 2.1. Chemical and Plant Material

The primary plant material used in this study was turmeric (*Curcuma longa*) rhizomes, which were sourced from a local agricultural supplier in Magetan, East Java, Indonesia. The rhizomes were selected based on visual quality and uniformity in maturity. Upon arrival, the turmeric was thoroughly washed with distilled water to remove any adhering soil or impurities. It was then sliced into thin sections of approximately 3–5 mm in thickness and subjected to drying using a dehydrator at 50 °C until a constant weight was achieved. The dried slices were subsequently ground into powder using a laboratory-scale grinder and sieved through 60 and 80 mesh screens to obtain a uniform particle size suitable for extraction. The following analytical-grade reagents were employed in the experimental procedures: choline chloride (≥99%), lactic acid (≥90%), standard curcumin (≥99% purity), acetonitrile (HPLC grade, ≥99%), and glacial acetic acid. All chemicals were procured from Sigma-Aldrich (St. Louis, MO, USA) and used without further purification. Ultrapure water (resistivity >18.2 MΩ·cm) produced by a Millipore purification system (Millipore, Burlington, MA, USA) was utilized in all solution preparations and analytical procedures.

### 2.2. Preparation of Turmeric and NADES Formulation

The *Curcuma longa* rhizomes used in this study were initially washed with distilled water to remove impurities, then sliced to a uniform thickness of approximately 3–5 mm. The sliced rhizomes were dried using a dehydrator at 50 °C until a constant weight was achieved, followed by pulverization using a laboratory-scale grinder. The resulting powder was sieved through 60 and 80 mesh screens to obtain a consistent particle size and stored in amber airtight containers to prevent degradation. The NADES was prepared by combining choline chloride (ChCl) and lactic acid in a molar ratio of 1:2. The mixture was stirred while being gently heated to 70 °C until a transparent and homogeneous solution was obtained. To reduce viscosity and improve mass transfer during the extraction process, ultrapure water was added to the NADES in varying proportions (20%, 25%, and 30% *v*/*v*), based on the experimental design. All solvents were prepared immediately prior to each extraction to ensure consistent physicochemical properties.

### 2.3. Extraction Procedures

#### 2.3.1. Ultrasound-Assisted Extraction (UAE)

**Figure 1:**
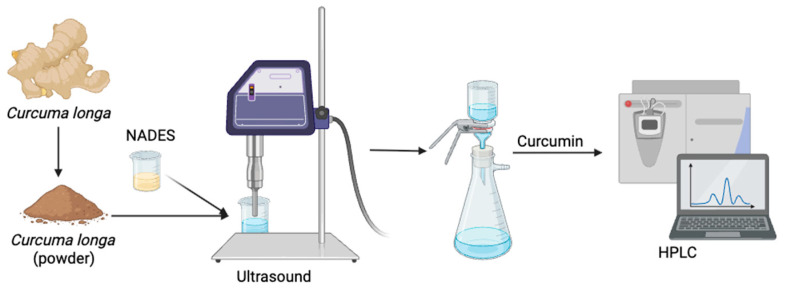
Schematic diagram of the curcumin extraction processes using UAE.

Ultrasound-assisted extraction (UAE) was conducted as a reference method based on previous work to evaluate the effectiveness of the proposed technique. In this method, 2.0 g of turmeric powder was mixed with NADES at a solid loading of 4% (*w*/*v*) and 20% (*v*/*v*) water content. The mixture was subjected to ultrasonic treatment using a 22 kHz ultrasonic probe system (Biobase Bioindustry, Jinan, China; model UCD-PO1) operated at 35 °C for 60 min with a 60% duty cycle. After extraction, the sample was centrifuged at 6000 rpm for 15 min, and the supernatant was filtered through a vacuum filtration unit to obtain the liquid extract. The curcumin content was subsequently analyzed using HPLC. The schematic method in UAE showed as Figure 1.

#### 2.3.2. Microwave-Ultrasound-Assisted Extraction (MUAE)

**Figure 2:**
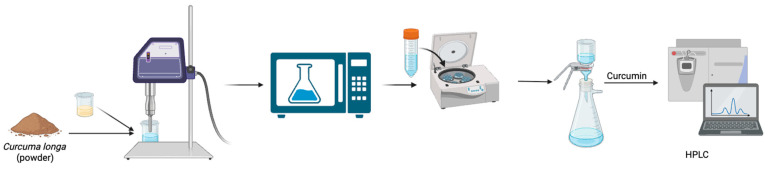
Schematic representation of the anti-solvent precipitation process used for curcuminoid separation and purification.

For the MUAE method, the same initial amount of 2.0 g turmeric powder was mixed with NADES at a fixed 20% (*v*/*v*) water content and varying solid loadings (4%, 6%, and 8% *w*/*v*). The mixture was first stirred to ensure uniform dispersion and then subjected to microwave pretreatment using a 400 W microwave oven (LG, Seoul, Republic of Korea; model MS2336GIB) for 1 min. This pretreatment aimed to disrupt the plant matrix and enhance the release of intracellular compounds. Following the microwave step, the sample underwent ultrasonic extraction using a 22 kHz ultrasonic probe at 35–45 °C for 60 min, maintaining a 60% duty cycle. After sonication, the extract was centrifuged at 6000 rpm for 15 min, and the supernatant was filtered through vacuum filtration. The resulting liquid extract was analyzed to determine the curcumin content via HPLC. The schematic representation of MUAE can be seen in Figure 2.

### 2.4. Separation and Purification of Curcuminoids

Following the extraction process, curcuminoids were separated from the NADES matrix using a precipitation method with water as an anti-solvent. The liquid extract was mixed with ultrapure water at a ratio of 1:20 (*v*/*v*) and incubated at 0 °C for 8 h to promote precipitation of curcuminoid solids. The mixture was then subjected to centrifugation at 6000 rpm for 15 min, and the resulting precipitate was isolated by vacuum filtration. The obtained solid was carefully collected, dried, and weighed to determine the total mass of curcuminoids recovered. A portion of the solid was dissolved in acetonitrile for high-performance liquid chromatography (HPLC) analysis to evaluate its curcumin content and purity. This separation method was designed to exploit the differences in polarity between the hydrophilic NADES solvent and the hydrophobic curcuminoid compounds, enabling efficient recovery and partial purification without the use of organic solvents.

### 2.5. Analytical Techniques

#### 2.5.1. Determination of Curcumin Content

The curcumin content in the extracted samples was quantified using high-performance liquid chromatography (HPLC). The analysis was conducted on a Shimadzu LC-40D XR system equipped with a UV/VIS detector (SPD-M40; Shimadzu, Kyoto, Japan) and a C18 column (4.6 mm × 250 mm, 5 μm). The mobile phase consisted of a mixture of acetonitrile and 2% acetic acid in distilled water at a ratio of 40:60 (*v*/*v*). The flow rate was set at 1.6 mL/min, the column temperature was maintained at 33 °C, and the detection wavelength was set to 425 nm. Each sample was analyzed in triplicate to ensure accuracy and reproducibility. The curcumin content was calculated using a calibration curve constructed from a series of standard curcumin solutions. The final concentration of curcumin in the extract was expressed as milligrams per gram of turmeric dry weight (mg/g). The determination of curcumin content can be calculated using Equation (1)

(1) $$CC\left(mg/g\right)=\frac{C\times N\times V}{W}$$

where *C:* concentration of curcumin in the extract (ppm); *N*: dilution factor; *V*: volume of extract (L); *W*: mass of turmeric powder (g).

#### 2.5.2. Determination of Purity and Recovery

The purity and recovery of curcuminoids were evaluated to assess the effectiveness of the anti-solvent precipitation process. Purity was determined both before and after separation to quantify the extent of purification achieved. Prior to separation, the curcumin concentration in the liquid extract was measured using HPLC. This value, along with the dilution factor and the density of the extract, was used to calculate the initial purity of curcuminoids in the solution. Following separation, the dried curcuminoid solid was weighed, and a known portion was redissolved in acetonitrile for HPLC analysis. The post-separation purity was calculated by dividing the mass of curcumin detected in the solid sample by the total mass of the redissolved sample. This allowed for an accurate estimation of the curcumin enrichment achieved through the precipitation step. The recovery percentage was calculated as the ratio of the total mass of curcuminoids recovered after precipitation to the theoretical mass of curcumin present in the original extract. This provided insight into the efficiency of the isolation process and helped identify potential losses due to filtration, solubility limitations, or incomplete precipitation. The purity content before separation can be calculated using Equation (2); in addition, the purity content after separation can be calculated using Equation (3), and recovery of Curcuminoid can be calculated using Equation (4)

(2) $$\%\;Purity\;Before\;Separation=\frac{C_{cur}^{0}\times N}{\rho_{eks}}\times 100\%$$

where C^0^_cur_: curcumin concentration in the liquid extract measured in HPLC (ppm); N: dilution factor; ρ_eks_: density of liquid extract (mg/L).

(3) $$\%\;Purity\;After\;Separation=\frac{C_{cur}\times V_{acn}}{m_{cur}}\times 100\%$$

where C_cur_: concentration of curcumin solids measured in HPLC (ppm); m_cur_: mass of curcumin solids sample for HPLC (mg); V_acn:_ volume of acetonitrile for HPLC dilution (L) and

(4) $$\%\;Recovery=\frac{m_{total}\times purity\;after\;separation(\%)}{C_{extract}\times V_{extract}}$$

where m_total_: total mass of curcuminoid solids (mg); C_extract_: extract concentration (ppm); V_extract_: volume of separation extract (L).

### 2.6. Experimental Design and Statistical Analysis

To evaluate the influence of solvent water content and solid loading on curcumin extraction efficiency, a central composite design (CCD) was employed as part of RSM using Design-Expert^®^ version 13 software. The CCD was selected due to its suitability for fitting second-order polynomial models and its effectiveness in optimizing process parameters with a minimal number of experimental runs. The two independent variables in this study were solvent water content (% *v*/*v*) and solid loading (% *w*/*v*). Each factor was varied at five levels, including axial, factorial, and center points, resulting in a total of 11 experimental runs, which were conducted in duplicate to ensure reproducibility. The experimental design matrix included both low and high extreme values as well as midpoints to accurately capture potential curvature in the response surface.

The response variable was the curcumin content expressed in mg/g of dry turmeric. The experimental data were analyzed using analysis of variance (ANOVA) to determine the significance of each factor and their interaction effects. Model adequacy was evaluated based on key statistical indicators, including the coefficient of determination (R^2^), adjusted R^2^, predicted R^2^, F-values, and *p*-values. A significance level of *p* < 0.05 was considered statistically significant. Additionally, model validation was performed by comparing the predicted values to actual experimental results to ensure the robustness of the optimization approach.

## 3. Result and Discussion

### 3.1. Influence of MUAE Process Parameters

The MUAE method demonstrated superior performance in extracting curcumin from *Curcuma longa* compared to the benchmark UAE method. By applying microwave irradiation for 1 min at 400 W prior to sonication, the method successfully disrupted turmeric’s plant cell matrix, facilitating solvent penetration without visible degradation of bioactive compounds. This thermal preconditioning, followed by ultrasound-induced cavitation at 22 kHz for 60 min, enabled effective mass transfer and metabolite release. The experimental results showed that the MUAE method achieved a maximum curcumin content of 40.72 ± 1.21 mg/g under optimized conditions, which is significantly higher than the UAE method that yielded 35.60 ± 2.35 mg/g. This corresponds to a 14.36% increase in extraction yield, demonstrating the efficacy of the MUAE method for enhancing bioactive compound recovery. Additionally, due to the higher optimal solid loading (8% *w*/*v*) used in the MUAE method versus 4% in UAE, a 50% reduction in solvent volume was achieved, improving the process sustainability. These results are summarized in Table 1, which presents the CCD-based response data for the tested factor levels.

In previous studies, propylene glycol or ethylene glycol has been reported as an effective solvent for curcumin extraction due to its high boiling point and polarity, facilitating solubilization of curcuminoids [25,26]. However, its synthetic origin, potential toxicity, and poor biodegradability pose limitations for pharmaceutical and nutraceutical applications. In contrast, the NADES used in this study, composed of choline chloride and lactic acid, offers a greener alternative with low toxicity and enhanced biodegradability. Compared to water and methanol, NADES-based extraction achieved up to 18% higher yields with 50% less solvent volume, highlighting efficiency and environmental benefits [22]. Another previous study found that a choline chloride–propylene glycol NADES (DES3) increased curcuminoid bioavailability by 450.4% and achieved peak plasma curcumin concentrations 6.93 times higher than ethanol extracts [27]. NADES extractions avoid toxic residues common in organic solvents, are biodegradable, and are compatible with food/pharma uses. Studies emphasize their low toxicity, biodegradability, and reusability, making them a greener option [28].

The integration of microwave and ultrasound energy in the MUAE method significantly outperformed conventional UAE in curcumin extraction, achieving a maximum curcumin content of 40.72 ± 1.21 mg/g under optimized conditions. This represents a 14.36% improvement over UAE (35.60 ± 2.35 mg/g), attributable to the synergistic enhancement of cell disruption and mass transfer mechanisms. Specifically, microwave pretreatment facilitated thermal disruption of plant tissues, increasing permeability, while ultrasound cavitation accelerated solvent penetration and compound release. Similar synergistic effects have been reported by [19], although their system employed MAE alone and used synthetic deep eutectic solvents, yielding comparatively lower curcumin content values and requiring greater solvent input. In contrast to studies utilizing NADES with only ultrasound or microwave activation, such as those by Jovanović et al. (2025) [22] and Rodsamai et al. (2024) [20], the present work uniquely combines both energy sources with NADES and systematically optimizes solid-to-solvent ratios, resulting in higher yields and better solvent economy. Furthermore, the 50% reduction in solvent usage in the MUAE system underscores its superior mass transfer efficiency and aligns with the principles of green extraction [22]. Previous works did not comprehensively evaluate the influence of solvent volume on process sustainability, highlighting a key innovation in this study. Importantly, the ability of the MUAE method to perform efficiently at higher solid loadings (8% *w*/*v*), as opposed to UAE’s limitation at 4%, demonstrates a practical advantage for industrial scaling. Higher solid content often results in solvent saturation and cavitation damping in conventional systems; however, the MUAE approach effectively mitigated these limitations, suggesting enhanced energy dispersion and mass transfer kinetics. These improvements are consistent with prior mechanistic theories but were not empirically confirmed in previous NADES-based curcumin extraction studies.

### 3.2. Optimization Using RSM

To optimize curcumin extraction using the MUAE method, a CCD was employed to model and evaluate the effects of two key independent variables: solvent water content (% *v*/*v*) and solid loading (% *w*/*v*). The resulting model aimed to predict the curcumin content as the response variable and determine the optimal operational conditions for maximum yield. The statistical analysis began with the sequential model sum of squares, presented in Table 2, which revealed that the quadratic model best fit the experimental data. This conclusion was supported by a significant *p*-value (*p* = 0.03) and an F-value of 8.37, indicating that the quadratic model captured the relationship between the variables more accurately than the linear or 2FI models.

To validate the model further, lack-of-fit analysis was performed (Table 3). The quadratic model showed the highest *p*-value (0.91) and the lowest lack-of-fit F-value (0.16), indicating no significant unexplained variation, which confirms the model’s adequacy for predicting curcumin yield.

The model summary statistics, provided in Table 4, also confirmed the robustness of the quadratic model. It yielded the highest adjusted R^2^ (0.96) and predicted R^2^ (0.94) values among all models tested, further supporting its selection for optimization.

The ANOVA results for the selected quadratic model are detailed in Table 5. Solvent water content (Factor A) showed a statistically significant influence on curcumin yield (*p* < 0.0001), while solid loading (Factor B) alone was not significant (*p* = 0.94). However, the interaction (AB) and the quadratic term for solid loading (B^2^) were significant, suggesting a nonlinear relationship affecting extraction efficiency.

The mathematical model derived from the regression analysis is as follows:

curcumin content (mg/g) = 73.67874 − 0.864657A − 5.12775B − 0.184425AB + 0.006589A^2^ + 0.813184B^2^

where A is solvent water content (%), B is solid loading (%).

The model’s predictive accuracy was further confirmed by comparing actual and predicted values, shown in Table 6. Most experimental values fell within 5% of predicted values, demonstrating excellent agreement.

**Figure 3:**
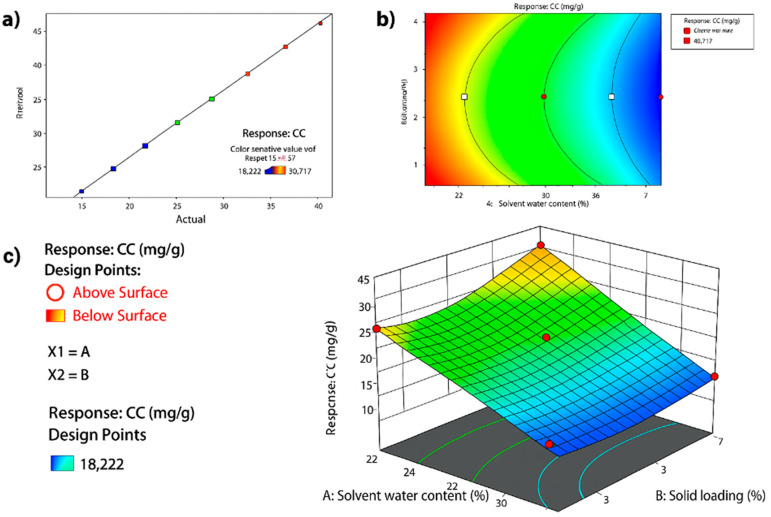
Plot of actual versus predicted curcumin content (**a**); Contour plot showing the interaction between solvent water content and solid loading on curcumin content (**b**); 3D response surface plot showing the effect of solvent water content and solid loading on curcumin content (**c**).

This model’s fit is visually illustrated in Figure 3a, where actual values cluster closely around the predicted linear regression line, confirming model accuracy. In Figure 3b, the 2D contour plot reveals optimal curcumin content at low water content (20%) and high solid loading (8%). The corresponding 3D surface plot in Figure 3c further highlights the interaction effect between the two variables.

The application of CCD and RSM in this study enabled a robust statistical evaluation of the interactive effects of solvent water content and solid loading on curcumin extraction efficiency. The optimized quadratic model (R^2^ = 0.98, adjusted R^2^ = 0.96) demonstrated superior fit and predictive capability, surpassing the performance metrics reported in earlier curcumin extraction models that lacked interaction terms or quadratic coefficients.

The model revealed that solvent water content had the most statistically significant effect on curcumin yield (*p* < 0.0001), consistent with prior findings in a previous study that noted a similar sensitivity of NADES viscosity and polarity to water dilution [29]. However, this study extended those observations by identifying a critical interaction between water content and solid loading (*p* = 0.04), as well as a quadratic response in solid loading (*p* = 0.01), neither of which were reported in previous single-variable optimizations. These insights provide a more comprehensive understanding of the parameter space for low-solvent utilization in extraction systems. Notably, the model predicted an optimal condition at 20% solvent water content and 8% solid loading conditions that were validated experimentally, with <5% deviation between predicted and actual curcumin content values. Such high predictive accuracy confirms the utility of CCD-RSM, not only for parameter screening but also for scalable process development.

### 3.3. Effect of Solvent Water Content on Curcumin Yield

**Figure 4:**
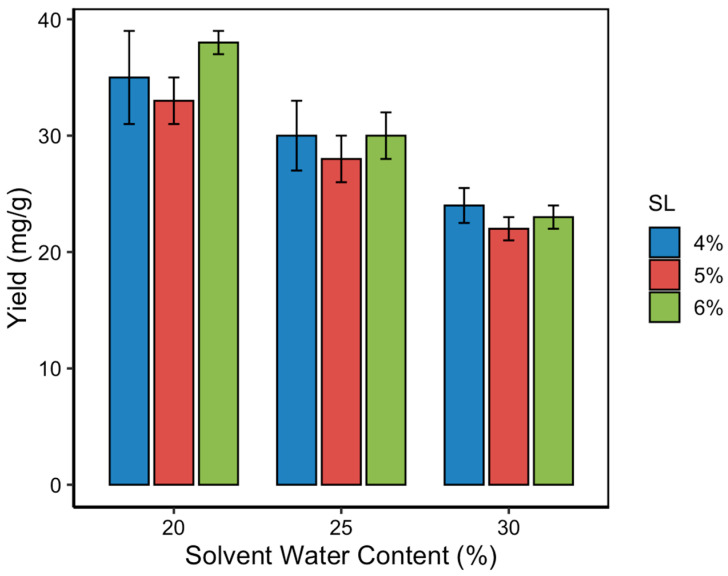
Effect of solvent water content on curcumin content at various solid loading levels.

The solvent water content in NADES significantly influences their viscosity, polarity, and capacity to interact with target compounds. In this study, three water content levels (20%, 25%, and 30% *v*/*v*) were evaluated to assess their impact on the curcumin content during MUAE extraction. As shown in Figure 4, curcumin yield decreased progressively with increasing water content, regardless of the solid loading level. The highest curcumin content was obtained at 20% water content, which is consistent with the RSM-predicted optimum. This result highlights that a moderate level of hydration improves mass transfer by lowering solvent viscosity, but excessive dilution can disrupt the hydrogen bonding network essential for NADES functionality. NADES systems rely on strong intermolecular hydrogen bonds between the hydrogen bond donor (lactic acid) and acceptor (choline chloride). When water content exceeds a critical threshold (typically > 25%), it dilutes these interactions, weakens solute–solvent affinity, and reduces the solvent’s ability to solubilize hydrophobic compounds like curcumin. This effect leads to decreased curcumin solubility and, consequently, lower extraction yields. The observed trend aligns with previous findings, in which small additions of water to NADES improved extraction performance up to a point [29], beyond which efficiency declined due to compromised supramolecular structure and reduced curcumin–NADES affinity.

The optimization of solvent water content and solid loading is crucial in maximizing curcumin yield [28,29,30]. This study identified 20% water content in the NADES and an 8% (*w*/*v*) solid loading as optimal, achieving a curcumin content of 40.72 ± 1.21 mg/g. This finding aligns with previous research, indicating that moderate water content reduces NADES viscosity, enhancing mass transfer without compromising solvent integrity [31,32,33,34]. Comparatively, studies utilizing higher water content reported diminished extraction efficiency, likely due to excessive dilution of the solvent system [35,36,37]. This result underscores the importance of balancing solvent composition to maintain optimal extraction conditions.

### 3.4. Effect of Solid Loading on Curcumin Yield

**Figure 5:**
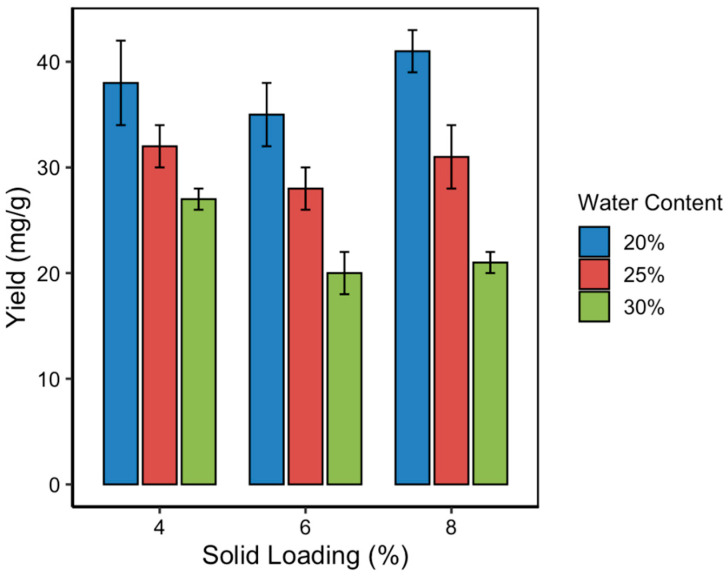
Effect of solid loading on curcumin content at different solvent water contents.

Solid loading, defined as the ratio of turmeric mass to solvent volume, is a critical parameter that influences extraction kinetics, mass transfer rates, and solvent saturation [38,39,40]. In this study, solid loading was varied at 4%, 6%, and 8% (*w*/*v*) to determine its effect on curcumin content in the MUAE system. As illustrated in Figure 5, the relationship between solid loading and curcumin yield exhibited a non-linear trend. Initially, increasing solid loading from 4% to 6% resulted in a decrease in curcumin content. However, further increasing the loading to 8% significantly enhanced the curcumin yield, surpassing even the 4% baseline. The highest yield (40.72 ± 1.21 mg/g) was recorded at 8% solid loading, particularly when paired with 20% solvent water content, confirming the optimal point predicted by the RSM model.

This unexpected rise in curcumin content at higher solid loading may be attributed to enhanced microwave heat distribution during the pretreatment step. At lower sample volumes, the rate of microwave energy absorption per unit mass is higher, potentially leading to more effective cell wall rupture and increased release of intracellular curcuminoids. Additionally, higher solid concentrations may enhance the concentration gradient between the plant matrix and the solvent, driving greater mass transfer. On the other hand, overly dense mixtures at solid loadings above 8% (not tested in this study) could lead to increased viscosity, reduced penetration of ultrasonic waves, and solvent saturation effects, potentially decreasing extraction efficiency. Hence, optimizing solid loading is crucial, not only for yield but also for maintaining practical flow and mixing conditions in scale-up applications.

The MUAE–NADES approach demonstrated superior performance compared to traditional extraction methods [23]. For instance, conventional Soxhlet extraction yielded lower curcumin content and required longer extraction times [41,42,43]. In contrast, our method achieved higher yields in significantly reduced timeframes, highlighting its efficiency. Additionally, the use of NADES as a green solvent aligns with sustainable extraction practices, offering an environmentally friendly alternative to organic solvents commonly used in traditional methods.

### 3.5. Comparative Analysis of MUAE Versus UAE: Yield Efficiency and Solvent Utilization

To validate the effectiveness of the MUAE method, its performance was directly compared to the conventional UAE technique. The comparison focused on two critical metrics: curcumin yield and solvent usage efficiency. The optimized MUAE process achieved a maximum curcumin content of 40.72 ± 1.21 mg/g, whereas the optimized UAE process yielded only 35.60 ± 2.35 mg/g under similar extraction conditions. This represents a 14.36% increase in extraction yield when using the MUAE method. The enhancement can be attributed to the synergistic effect of microwave pretreatment, which disrupts plant cell structures and facilitates solvent diffusion, followed by ultrasound-induced cavitation that accelerates compound release.

**Figure 6:**
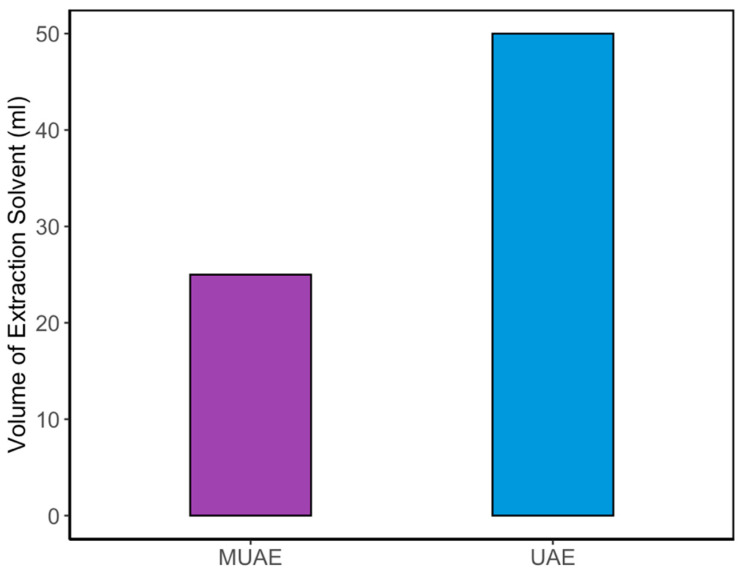
Comparison of solvent volume usage between UAE and MUAE methods.

In addition to higher yield, the MUAE method significantly improved solvent efficiency. Under optimal conditions, MUAE required only half the amount of NADES solvent compared to the UAE method. This improvement stems from the ability of MUAE to operate effectively at a higher solid loading (8% *w*/*v*), whereas UAE was optimized at 4% *w*/*v*. As a result, the MUAE process reduced solvent usage by 50% while delivering a superior product yield. These findings are clearly illustrated in Figure 6, which compares the total solvent volume required for each method to achieve their respective optimum yields. The MUAE method not only enhances curcumin recovery but also supports the principles of green chemistry by minimizing solvent consumption and energy use making it a more sustainable and scalable option for industrial applications.

The combination of microwave and ultrasound energies in the MUAE method resulted in a synergistic enhancement of curcumin extraction. Microwave irradiation facilitates rapid heating and cell disruption, while ultrasound promotes cavitation, improving solvent penetration and mass transfer [44,45,46,47]. This synergy leads to higher extraction efficiency compared to the application of either technique alone. Previous studies have reported similar synergistic effects in the extraction of bioactive compounds, supporting the efficacy of combining these technologies [45]. These findings contribute to this body of knowledge by demonstrating the effectiveness of MUAE in curcumin extraction.

### 3.6. Curcuminoid Separation Efficiency and Purity Improvement

Following extraction, a critical step in producing high-purity curcuminoids is their effective separation from the NADES matrix. In this study, an anti-solvent precipitation method using ultrapure water was employed to recover curcuminoids from the MUAE extract. The process involved the addition of water at a 1:20 (*v*/*v*) ratio followed by incubation at 0 °C for 8 h, allowing the hydrophobic curcuminoids to precipitate due to their reduced solubility in the diluted, now highly polar, solvent mixture. The separation performance was evaluated by comparing curcumin purity before and after precipitation and calculating the recovery efficiency. Before separation, the curcumin purity in the liquid extract was only 0.31%, while after precipitation, the purity increased substantially to 20.54%, as determined by HPLC analysis.

This dramatic improvement indicates the success of water-induced polarity shift in breaking the hydrogen bonding interactions between NADES and curcuminoids. A similar water-based anti-solvent approach [48], in which curcuminoid recovery reached up to 39% using n-hexane, demonstrating the potential of phase separation techniques for purifying turmeric extracts. In the current study, despite the notable increase in purity, the recovery rate of curcuminoids was relatively modest at 21.49%. This limited recovery may be attributed to losses during filtration, poor sedimentation of nanoparticles, or retention of curcuminoids on filter surfaces issues was also encountered in other studies involving NADES-based extractions.

**Figure 7:**
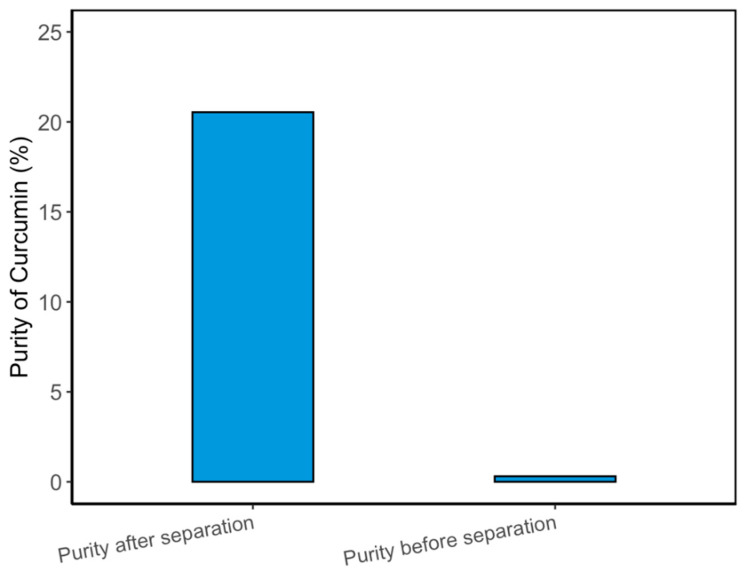
Comparison of curcumin purity before and after separation using anti-solvent precipitation.

Additionally, the formation of extremely fine particles may have allowed some curcuminoids to pass through the filter media. As shown in Figure 7 the purity improvement demonstrates the effectiveness of the precipitation technique in isolating curcuminoids from green solvents without resorting to organic solvents or complex purification systems. Future process optimization, such as improved mixing, cooling control, or membrane-based filtration, may enhance the recovery rate and support scale-up viability.

### 3.7. Mechanistic Insights: Mass Transfer, Cavitation, and Viscosity Effects

The success of the MUAE process is underpinned by several key physicochemical mechanisms that act synergistically to enhance curcumin extraction: microwave-induced matrix disruption, ultrasonic cavitation, and modulation of NADES viscosity through water addition. Understanding these mechanisms is essential for process optimization and scale-up. Microwave pretreatment disrupts plant cell structures by rapidly heating intracellular water, causing pressure build-up and rupture of cell walls. This disruption enhances the accessibility of curcuminoids to the solvent, particularly in samples with low to moderate moisture content [20,49,50]. The short duration (1 min at 400 W) used in this study was sufficient to soften the turmeric matrix without degrading thermolabile curcuminoids, which are known to begin degrading above 80–85 °C.

Ultrasound energy further amplifies extraction efficiency through cavitation as the formation, growth, and collapse of microbubbles generate localized shear forces [51,52]. These mechanical effects improve mass transfer by enhancing solvent penetration into the disrupted plant tissue and accelerating the release of intracellular compounds. However, excessive solid loading or solvent viscosity can suppress cavitation, reducing ultrasonic effectiveness. Thus, the optimal 8% solid loading achieved in this study reflects a balance between maximizing compound concentration and maintaining fluidity for effective wave propagation.

Viscosity plays a central role in solvent performance, particularly for NADES, which are inherently more viscous than conventional solvents [53,54]. Water was added to NADES to decrease viscosity and improve diffusion rates. At 20% water content, NADES maintained sufficient hydrogen bonding to solubilize curcuminoids while achieving lower resistance to mass transfer. Beyond 25% water content, the weakening of NADES’s hydrogen bond network led to decreased curcumin solubility, as observed in reduced yields. This result is in line with the previous study which determined that 25% of water content generally provides optimal stability. These mechanistic insights not only validate the parameter optimization obtained through RSM but also reinforce the MUAE method’s advantages over conventional extraction techniques. The combination of physical cell rupture, enhanced mass transfer, and tailored solvent viscosity creates a powerful extraction environment capable of delivering high yields with minimal environmental impact.

### 3.8. Sustainability and Industrial Relevance

The growing demand for clean-label, natural, bioactive ingredients such as curcumin has intensified the need for extraction processes that are not only efficient but also environmentally sustainable. This study demonstrates that the MUAE method, when combined with NADES, offers a compelling green alternative to conventional extraction techniques. From a sustainability perspective, the MUAE method aligns with the 12 principles of green chemistry, particularly in its reduction of solvent volume, lower energy input, and avoidance of toxic organic solvents. The optimized process achieved a 14.36% higher yield compared to UAE while also reducing solvent usage by 50%, thereby lowering the environmental footprint of the extraction process.

The use of NADES composed of choline chloride and lactic acid, both of which are biodegradable and derived from renewable resources, further enhances the environmental compatibility of the process. Unlike organic solvents such as ethanol or acetone, NADES pose minimal risk in terms of toxicity, flammability, and waste disposal. Moreover, the precipitation-based recovery of curcuminoids using only water as an anti-solvent minimizes secondary waste and eliminates the need for additional purification solvents, supporting waste reduction and process simplicity. From an industrial standpoint, the MUAE technique offers several advantages for scaling up curcumin production. The short extraction time, high yield at low solvent-to-solid ratios, and ambient-to-mild processing temperatures make the method economically attractive and technically feasible. The process is also modular, allowing for adaptation to continuous or semi-continuous flow systems, particularly with the integration of scalable microwave and ultrasound reactors.

The MUAE–NADES method presents a scalable and sustainable approach for curcumin extraction. Its high efficiency, reduced solvent usage, and shorter extraction times make it attractive for industrial applications. Moreover, the use of green solvents aligns with environmental regulations and consumer demand for eco-friendly products. Implementing this method in the industry could lead to cost savings, reduced environmental impact, and improved product quality. Future research should focus on scaling up the process and exploring its applicability to other bioactive compounds.

However, to transition from laboratory to industrial scale, further work is needed to optimize the recovery and reuse of NADES, improve precipitation yield, and ensure regulatory compliance for food and pharmaceutical applications. Additionally, Life Cycle Assessment (LCA) and Techno-Economic Analysis (TEA) are recommended in future studies to quantify the broader sustainability benefits and operational costs compared to conventional systems.

Lastly, the future recyclability of NADES is a key consideration for sustainable process development. Following curcuminoid separation via anti-solvent precipitation, the NADES rich aqueous phase can be recovered and recycled using techniques such as rotary evaporation or vacuum-assisted distillation to remove excess water and restore the original solvent properties. Several studies have shown that NADES systems, particularly those based on choline chloride and organic acids, can retain their physicochemical characteristics and extraction efficiency over multiple cycles with minimal degradation [19,54]. Additionally, membrane filtration and ion exchange have been proposed as green alternatives for solvent regeneration. Future work will focus on evaluating the stability, compositional integrity, and efficiency of NADES across successive reuse cycles to establish a closed-loop system for industrial application.

### 3.9. Recommendation for the Future Research Direction

To further advance the potential of the MUAE–NADES system for industrial-scale curcumin extraction, several key research directions are proposed. First, while the anti-solvent precipitation method significantly improved curcumin purity, the overall recovery rate remained modest at 21.49%. This suggests that a considerable portion of curcuminoids may be lost during separation, either due to incomplete precipitation or filtration inefficiencies. Future studies should explore advanced separation techniques such as membrane filtration, dynamic crystallization, or electro-assisted precipitation, which may enhance recovery while maintaining product purity. Second, the recyclability and regeneration of NADES solvents must be thoroughly evaluated. Although NADES are considered green and biodegradable, their economic and environmental viability at industrial scale hinges on their reusability. Investigating solvent stability, component degradation, and recovery efficiency after multiple extraction cycles will be critical for designing a circular extraction process.

In addition, scaling up the MUAE process from laboratory to pilot or industrial scale presents technical challenges that must be addressed. Further research is needed to integrate microwave and ultrasound reactors into continuous or semi-continuous systems, where factors such as heat distribution, energy efficiency, and solvent dynamics under flow conditions require optimization. Moreover, comprehensive sustainability assessments should be conducted through Life Cycle Assessment (LCA) and Techno-Economic Analysis (TEA). These analyses will provide quantitative insights into the environmental impact, carbon footprint, and cost-efficiency of the MUAE–NADES system compared to conventional solvent-based extraction methods, strengthening its case for commercial adoption.

Expanding the application of this low-solvent utilization and extraction platform to other phytochemicals may also prove valuable. The MUAE–NADES system has potential for extracting a wide range of bioactives, including flavonoids, polyphenols, and essential oils from diverse plant sources, enabling the development of integrated biorefinery approaches. Finally, to support regulatory approval and commercial application in the food and pharmaceutical sectors, further studies on the toxicological safety and biocompatibility of NADES components are essential. Establishing compliance with international standards will ensure safe utilization of NADES-extracted curcumin in consumer products. By addressing these research gaps, future work can transform MUAE–NADES into a fully sustainable, high-yield, and industry-ready platform for natural product extraction.

## 4. Conclusions

This study demonstrated that the combination of MUAE using NADES is an effective, and scalable method for extracting curcumin from *Curcuma longa*. The optimal extraction conditions were achieved at 20% solvent water content and 8% solid loading, resulting in a maximum curcumin content of 40.72 ± 1.21 mg/g, representing a 14.36% improvement over the conventional UAE method. Furthermore, the MUAE method reduced solvent consumption by 50%, supporting the principles of green chemistry (low chemical use) and offering a more sustainable solution for natural product recovery. The statistical modeling using RSM with a quadratic model proved highly effective for process optimization, with excellent agreement between predicted and actual values. The curcuminoid separation via anti-solvent precipitation increased purity from 0.31% to 20.54%, although the recovery rate remained relatively low at 21.49%. Overall, this study confirms the potential of MUAE combined with NADES as a robust and environmentally friendly approach for curcumin extraction, paving the way for its application in nutraceutical, pharmaceutical, and food industries.

## Figures and Tables

**Table 1:** RSM experiment matrix with CCD.

No.	Solvent Water Content (%)	Solid Loading (%)	Curcumin Content (mg/g)
1	20	4	36.75 ± 5.73
2	20	6	35.23 ± 1.65
3	20	8	40.72 ± 1.21
4	25	4	29.97 ± 2.79
5	25	6	29.51± 2.10
6	25	6	26.41± 1.85
7	25	6	26.08± 1.70
8	25	8	29.66 ± 3.78
9	30	4	24.32 ± 0.42
10	30	6	18.22 ± 2.30
11	30	8	20.91 ± 0.42

**Table 2:** Sequential model sum of squares for model selection.

Model Comparison	Sum of Squares	df	Mean Square	F-Value	*p*-Value
Mean vs. Total	9180.43	1	9180.43	—	—
Linear vs. Mean	404.3	2	202.15	30.99	0
2FI vs. Linear	13.61	1	13.61	2.47	0.16
Quadratic vs. 2FI	29.71	2	14.85	8.37	0.03
Cubic vs. Quadratic	0.38	2	0.19	0.07	0.94
Residual	8.5	3	2.83	—	—

**Table 3:** Lack-of-fit test for model adequacy.

Source	Sum of Squares	df	Mean Square	F-Value	*p*-Value
Linear	45.05	6	7.51	2.1	0.36
2FI	31.45	5	6.29	1.76	0.4
Quadratic	1.74	3	0.58	0.16	0.91
Cubic	1.36	1	1.36	0.38	0.6
Pure Error	7.14	2	3.57	—	—

**Table 4:** Model summary statistics.

Model	Std. Dev.	R^2^	Adjusted R^2^	Predicted R^2^	PRESS
Linear	2.55	0.89	0.86	0.74	117.82
2FI	2.35	0.92	0.88	0.65	159.25
Quadratic	1.33	0.98	0.96	0.94	26.29
Cubic	1.68	0.98	0.94	0.59	185.9

**Table 5:** ANOVA for curcumin content using the quadratic model.

Source	Sum of Squares	df	Mean Square	F-Value	*p*-Value
Model	447.62	5	89.52	50.43	<0.0001
A—Water Content	404.29	1	404.29	227.76	<0.0001
B—Solid Loading	0.01	1	0.01	0.01	0.94
AB	13.61	1	13.61	7.66	0.04
A^2^	0.07	1	0.07	0.04	0.85
B^2^	26.8	1	26.8	15.1	0.01
Residual	8.88	5	1.78	—	—
Lack of Fit	1.74	3	0.58	0.16	0.91
Pure Error	7.14	2	3.57	—	—
Total	456.49	10	—	—	—

**Table 6:** Comparison of actual and predicted curcumin content.

Water Content (%)	Solid Loading (%)	Actual Curcumin Content (mg/g)	Predicted Curcumin Content (mg/g)	Error (%)
20	4	36.75 ± 5.73	36.77	0.04%
20	6	35.23 ± 1.65	35.4	0.47%
20	8	40.72 ± 1.21	40.53	0.45%
25	4	29.97 ± 2.79	30.24	0.89%
25	6	29.51 ± 1.89	27.02	8.41%
25	6	26.41 ± 1.89	27.02	2.32%
25	6	26.08 ± 1.89	27.02	3.61%
25	8	29.66 ± 3.78	30.32	2.22%
30	4	24.32 ± 0.42	24.04	1.16%
30	6	18.22 ± 2.30	18.98	4.16%
30	8	20.91 ± 0.42	20.43	2.29%

## Data Availability

The datasets used and/or analyzed during the current study available from the corresponding author on reasonable request.
